# The Use of Oral Anticoagulation Is Not Independently Associated with Mortality in Frail Older Patients with Repeated Falls

**DOI:** 10.3390/jcm12237388

**Published:** 2023-11-29

**Authors:** Lennaert A. R. Zwart, Jeske J. Walgers, Martin E. W. Hemels, Tjeerd Germans, Joris R. de Groot, René W. M. M. Jansen

**Affiliations:** 1 Department of Geriatric Medicine, Northwest Clinics, 1815 JD Alkmaar, The Netherlands; 2 Department of Geriatric Medicine, Dijklander Hospital, 1624 NP Hoorn, The Netherlands; 3 Department of Cardiology, Rijnstate Hospital, 6815 AD Arnhem, The Netherlands; mhemels@rijnstate.nl; 4 Department of Cardiology, Radboud UMC, 6525 GA Nijmegen, The Netherlands; 5 Department of Cardiology, Northwest Clinics, 1815 JD Alkmaar, The Netherlands; t.germans@nwz.nl; 6 Department of Cardiology, Amsterdam UMC, 1105 AZ Amsterdam, The Netherlands; j.r.degroot@amsterdamumc.nl

**Keywords:** anticoagulants, atrial fibrillation, frail elderly, mortality

## Abstract

Background: Particularly in frail patients, anticoagulation may be underused because of the fear of bleeding. Objective: To determine whether the use of antithrombotic medication is an independent risk factor for mortality in frail elderly with repeated falls. Methods: All patients aged 65 years or older at the Fall and Syncope Clinic were eligible. Frailty was calculated with a Frailty Index (FI) based on the accumulation of deficits model. Risks were calculated with a cox regression analysis, adjusted for age, sex, and Frailty Index. Results: 663 patients were included in this analysis. The median age was 80 years, 438 were women (66%), 73% had polypharmacy, and 380 patients (57%) had cognitive impairment. The mean FI was 0.23 (sd 0.09), 182 patients were moderately frail (27.5%), and 259 (39.1%) were severely frail. A total of 140 (21%) used oral anticoagulation and 223 (34%) used antiplatelet agents. A total of 196 patients (29.6%) died during follow-up. In the adjusted cox regression model, the use of neither antiplatelets nor anticoagulation was associated with mortality. A strong association was found with frailty (HR 74.0, 95% CI 13.1–417.3) and a weak association with age (HR 1.05, 95% CI 1.03–1.08). A lower risk of mortality was seen in women (HR 0.5, 95% CI 0.3–0.6). Conclusions: In this cohort of frail older patients, there was no independent association between the use of antithrombotic medication and mortality. A strong association with mortality was found with frailty, a weak association was found with age, and a lower mortality risk was found in women. Our data indicate that the fear of bleeding or increased mortality in frail patients with an indication for oral anticoagulation may be unjustified.

## 1. Introduction

The most common indication for oral anticoagulation (OAC) in older people is atrial fibrillation (AF) [1]. In the coming decades, the prevalence of AF among people of 65 years and older is expected to roughly double, mostly with new cases in those aged over 80 years [1]. Patients with AF of 80 years and older form a high-risk group for both stroke and major bleeding [1,2] but also are more likely to be frail [3,4,5]. Frailty is defined as a clinical state in which patients have diminished functionality in different domains, such as physical and cognitive functioning and mobility, leading to a higher risk of adverse outcomes in general [6,7,8]. Frailty is a heterogeneous syndrome and can be difficult to operationalize because consensus on a clear and definite definition is lacking [6,7]. Many different screening or assessment tools have been developed, with or without the need of validation in larger cohorts, and with great heterogeneity among the tools, making a comparison of frailty between cohorts complicated [9,10,11]. A comprehensive geriatric assessment-based Frailty Index could overcome these issues, but a disadvantage is that it cannot easily be applied at the bed-side [7,12].

The ESC-EHRA EORP-AF General Long-Term Registry recently performed a thorough analysis of frailty among patients with AF, utilizing a 40-item Frailty Index [13]. This index found that the vast majority of AF patients are either pre-frail or frail (>80%), and increasing frailty was associated with a higher risk for all adverse outcomes investigated [13]. OAC lowered the risk of all adverse outcomes, except in patients with extremely high frailty scores. A large retrospective study using data from the Korean National Health Insurance Service database has similar outcomes [14]. The authors assessed the level of frailty of AF patients utilizing the Hospital Frailty Risk Score and found that patients on OAC had a lower risk of ischemic stroke (hazard ratio 0.91, 95% CI 0.86–0.97) and cardiovascular death (hazard ratio 0.52, 95% CI 0.49–0.55), but no difference was observed for major bleeding (hazard ratio 1.02, 95% CI 0.95–1.10) [14]. Furthermore, compared to warfarin, patients on direct oral anticoagulation (DOAC) had an even lower risk of all adverse outcomes studied [14]. In frail patients, physicians are concerned of an elevated risk of oral anticoagulation-related major bleeding, which, in the past, has led to undertreatment with or discontinuation of oral anticoagulation [1,15,16]. A study in the Netherlands among geriatric patients showed a very low rate of OAC use for AF of 58% in 2004 [17], but recently published results on geriatric outpatients show that 87% of geriatric patients with AF were treated with OAC [5].

Frailty is associated with adverse events, and indeed a recent meta-analysis shows that frail AF patients have a much higher risk of complications and mortality than robust patients with AF [6,7,16,18,19]. Physicians’ concerns on prescribing OAC in this group, balancing stroke prevention and anticoagulation related major bleeding, ensues from the primary principle of ‘do no harm’. The important question that remains, however, is not if frail patients have higher risks of adverse outcomes than robust patients, but what the added risk of OAC use is on top of the high residual risk that frail patients already possess. In other words, does the use of OAC cause harm? Ideally, this question should be addressed in a randomized clinical trial. The feasibility of performing such a trial can be questioned, as frailty forms a heterogenous clinical syndrome, introducing a variety of confounders and competing risks associated with co-morbidities. Consequently, to address this issue in a randomized trial, a very large cohort would be needed. Frail patients might be difficult to reach and motivate to participate, and furthermore, conducting a placebo versus OAC trial is not an ethical approach in patients at high risk for stroke. Therefore, at the moment, for this group, it is important to also consider prospective cohorts and cross-sectional studies for clinical decision making.

A sub-analysis of the randomized ENGAGE-AF TIMI 48 study by Steffel and colleagues showed that patients on oral anticoagulation with an increased risk of falling have a higher risk of major bleeding and clinically relevant non-major bleeding, fatal bleeding, and all-cause death but not intracranial hemorrhage or ischemic events [20]. The authors also found that these patients at risk of falling were considerably older (median age of 77 versus 72 years) and had a higher prevalence of multi-morbidity. Based on their definition of being at risk of falls, it is very likely that this sub-group was much frailer than those without a risk of falling [20]. Our recent analysis showed that within a cohort of frail patients, either the use of antiplatelet agents or oral anticoagulation significantly increases the risk of major bleeding [4]. However, despite their frailty, the risk of major bleeding per 100 treatment years of anticoagulation was similar to that in the large RCTs, in which frail patients were underrepresented [4]. Whether the use of anticoagulation or antiplatelet agents are independently associated with mortality among frail patients is less clear and is the focus of this analysis [1,19,20]. Here, we investigate the relationship of the use of OAC or antiplatelet agents with mortality, within a frail cohort, containing patients with and without AF.

## 2. Methods

We used data from the Fall and Syncope Registry of the Northwest Clinics, Alkmaar, the Netherlands. This registry contains data on all patients who underwent a comprehensive geriatric assessment (CGA) at the Fall and Syncope Clinic since November 2011. Details on the Fall and Syncope Clinic have been published previously [21]. Only patients with repeated, unexplained falls are evaluated at the Fall and Syncope Clinic; other patients who have experienced falls are assessed at the regular outpatient clinic and not included in the cohort or this analysis. The CGA includes a medical, neurological, psychological, functional, and cognitive evaluation. All patients gave written informed consent for the use of their medical record and the Ethical Board approved the Fall and Syncope Registry. To determine frailty, a Frailty Index based on the accumulation of deficits model was calculated [7,22]. The Frailty Index consists of 42 items: 29 somatic items, 9 functional items, and 4 cognitive items. Factors in the Frailty Index can be found in Supplementary Table S1. All items are scored as 0 points if absent and as 1 if present. The index is calculated as the ratio between present items and the total number of items, and ranges from 0 to 1. Patients are considered moderately frail if they have a score of 0.18 or higher and severely frail if they have a score of 0.25 or higher [4,5,22]. The index is designed to contain all factors associated with adverse outcomes such as unplanned hospitalization, major cardiovascular events, and mortality [7,8,11]. Although AF is strongly associated with those outcomes as well, it is not incorporated into the Frailty Index used in this analysis to avoid collinearity with the use of OAC [1].

For this analysis, follow-up data on mortality were collected from the hospital records and general practitioners offices for the period between November 2011 and May 2020. Patients of 65 years and older who visited the Fall and Syncope Clinic between November 2011 and February 2020 were included in this analysis. If patients visited the Fall and Syncope Clinic more than once, the first visit was used for this analysis. Calculations were made in IBM SPSS Statistics version 20. Baseline characteristics between patients with or without antithrombotic medication were analyzed, and associations between baseline characteristics and mortality were explored with a univariable binary logistic regression analysis. The association of antithrombotic medication and other patient characteristics with mortality was analyzed with regression analysis, the calculation of Hazard Ratio’s (HR), and confidence intervals. To correct the risk of mortality for time, a cox regression survival analysis was performed. The Frailty Index contained all possible (known) confounders for this cohort, and adjustments were applied for age, sex, and the Frailty Index as a continuous variable. The number of AF patients without OAC was expected to be very low, but as a descriptive analysis, a cox regression survival analysis was performed in patients with AF with and without OAC.

## 3. Results

There were 701 visits to the FSC between November 2011 and February 2020; in 670 visits, the patient gave informed consent for participation in the registry. A total of 7 patients visited the Fall and Syncope Clinic twice, leading to the inclusion of 663 patients in this analysis. The majority of patients used antithrombotic medication (363 patients, 54.7%); 144 patients used OAC (21.7%, 19 patients used direct oral anticoagulation and 125 used vitamin K antagonists), and 223 patients used antiplatelet agents (33.6%). Of the entire cohort, 196 patients died (29.6%), with a median time between inclusion and death of 2 years and 8 months.

**Figure 1:**
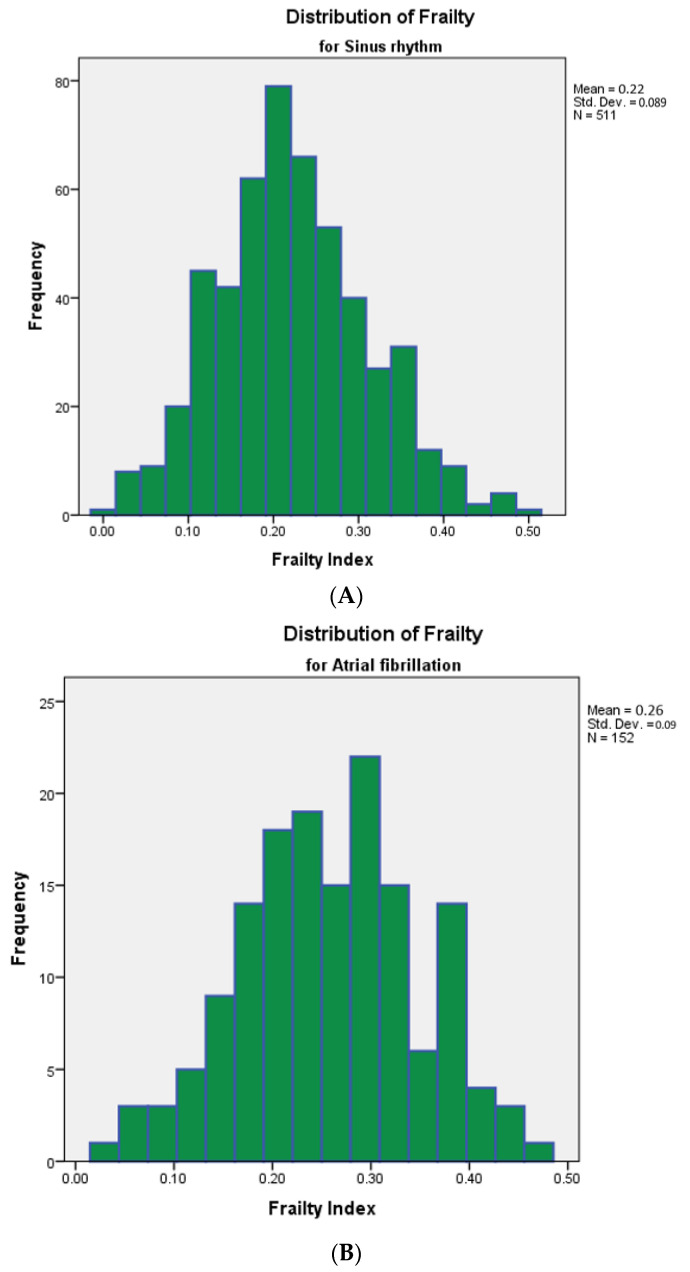
(**A**) Distribution of frailty in patients with sinus rhythm. (**B**) Distribution of frailty in patients with atrial fibrillation.

The median age was 80 ± 6.5 years, 438 were women (66.1%), the majority had polypharmacy (73% of patients used 5 prescription drugs or more; the median number of medication was 7, sd 3.8), and 380 patients (57.3%) showed signs of cognitive impairment on the Montreal Cognitive Assessment (MoCA) [23]. Of the 139 patients known with atrial fibrillation, 114 used OAC (82.0%), and OAC was initiated in 5 out 15 new cases of atrial fibrillation (33.3%). The mean FI was 0.23 ± 0.09; 222 patients (33.5%) were robust, 182 patients (27.5%) were moderately frail, and 259 (39.1%) were severely frail. The baseline characteristics of the entire cohort were divided between patients without antithrombotic medication and those using either antiplatelet agents or oral anticoagulation, as described in Table 1. Figure 1A,B show the distribution of frailty of patients with and without AF. For both patient groups, frailty is normally distributed, but patients with AF have a significantly higher Frailty Index (0.22 for patients in sinus rhythm and 0.26 for patients with AF, *p* < 0.001). Supplementary Figure S1 illustrates that the majority of patients with AF are severely frail, 23% are robust, 24% are moderately frail, and 53% are severely frail.

Patients using either antiplatelets or OAC at baseline (including those in which OAC was initiated because of newly diagnosed AF, 5/15 patients), were older, had higher morbidity, used more prescription drugs, and had a higher dependence on others for daily tasks of living, cumulating in a large difference in the Frailty Index. Patients on oral anticoagulation more frequently had AF (81.9%, *p* < 0.01) but also increased heart failure (25.7%, *p* < 0.01), whereas patients on antiplatelet agents more often had a history of stroke (43.0%, *p* < 0.01) or ischemic heart disease (42.5%, *p* < 0.01). The prevalence of cognitive impairment was not different between the groups.

The median follow-up time was 45 months (ranged from 1 to 101 months). Causes of death are described in Table 2. Mortality was 23.7% for patients not using antithrombotic therapy, 30.9% for patients using antiplatelets, and 37.1% for patients on oral anticoagulation. Outcomes of the crude univariable regression analysis show that various factors were associated with mortality, as shown in Table 3.

The strongest associations were found for a reduced creatinine clearance of 15 to 30mL/min (HR 9.0, 95% CI 2.8–28.7, *p* < 0.01), severe frailty (HR 3.5, 95% CI 2.3–5.5, *p* < 0.01), and heart failure (HR 3.4, 95% CI 2.0–5.7, *p* < 0.01). In univariable analysis, also the use of antiplatelets or OAC showed a significant association but of a smaller magnitude (HR 1.6, 95% CI 1.0–2.3, *p* = 0.03 and HR 2.1, 95% CI 1.4–3.3, *p* > 0.01, respectively). Of the geriatric features, frailty, post prandial hypotension, parkinsonism, gait disturbance, dependence in tasks of daily living (activities of daily living (ADL) and instrumental activities of daily living (iADL)), and scoring below 26 points on the Mini Mental State Examination (MMSE) were associated with mortality.

**Figure 2:**
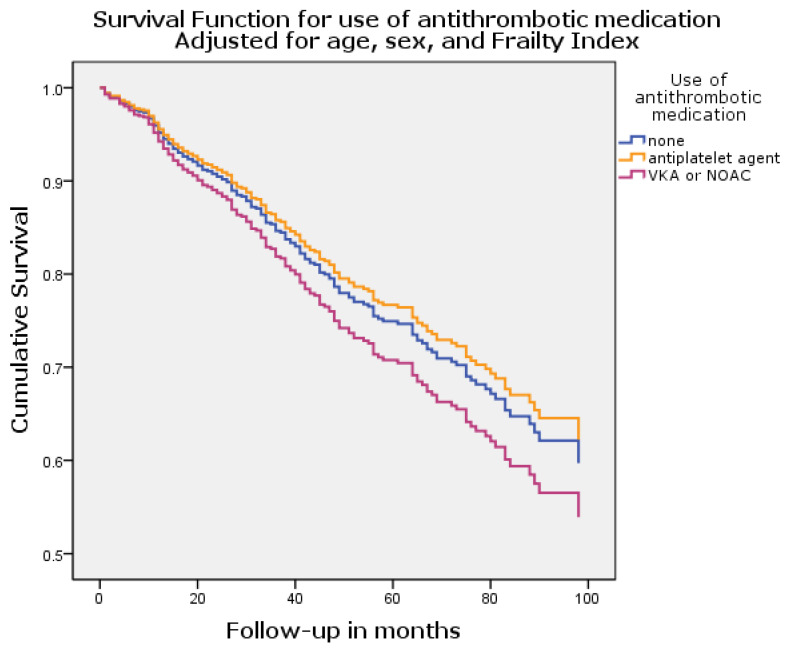
Survival curve adjusted for age, sex, and Frailty Index, per category of antithrombotic drug.

**Figure 3:**
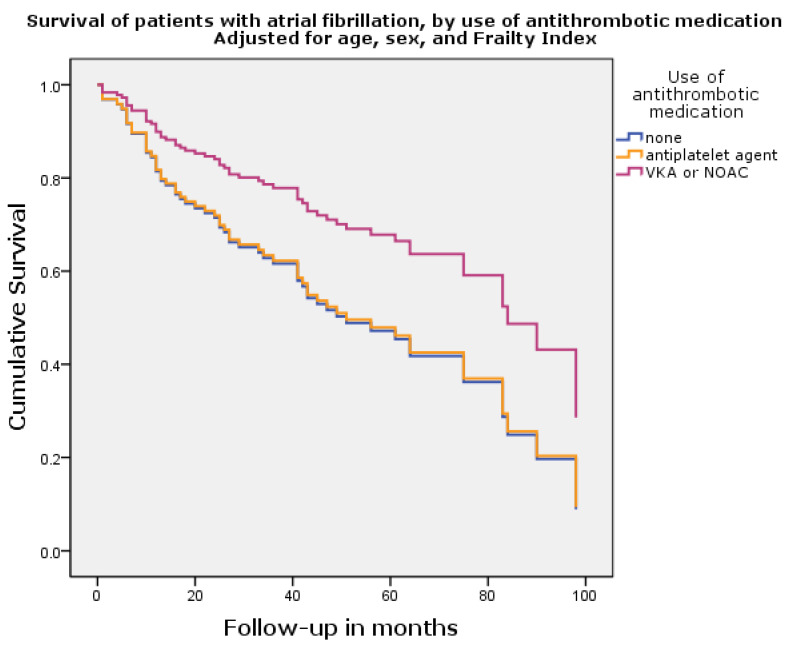
Survival curve of patients with atrial fibrillation only.

Figure 2 shows the survival curve based on the cox regression analysis adjusted for age, sex, and Frailty Index. Of note, to avoid collinearity, the Frailty Index does not include AF as a factor but functions as a composite of somatic, cognitive, functional, and social risk factors. The adjusted HRs are described in Table 4. After adjustment, antithrombotic medication was not significantly associated with mortality. The Frailty Index, however, remained strongly associated with mortality (HR 74.0, 95% CI 13.1–417.3, *p* < 0.001), as well as age (HR 1.05, 95% 1.03–1.08, *p* < 0.001) being associated with mortality. A lower risk of mortality was seen in women (HR 0.5, 95% CI 0.3–0.6, *p* < 0.001). In patients with AF, there was a numerical survival benefit for AF patients using OAC, as displayed in Figure 3. After adjusting for age, sex, and Frailty Index, the use of OAC showed a trend toward a lower risk of mortality, but this did not reach statistical significance (HR 0.52, 95% CI 0.21–1.25, *p* = 0.14). Also, sex was not significantly associated with mortality any longer (HR 1.67, 95% CI 0.99–2.82, *p* = 0.06), but age remained weakly associated (HR 1.05, 95% CI 1.01–1.09, *p* = 0.03).

## 4. Discussion

In this study, the association between the use of antiplatelet agents, OAC, and mortality within frail patients who frequently fall was analyzed. The strengths of this study are that the majority of the cohort was frail (66.5%) and mortality was high (29.6% during a median follow-up of 45 months). In addition to frailty, the prevalence of cognitive disorders was higher than in other studies including patients with AF, such as the EAST-AFNET 4 Trial (>55% in our study, versus 43.5% in the EAST-AFNET 4 trial) [24]. The use of either antiplatelet agents or oral anticoagulation was not found to be independently associated with mortality in frail older patients who repeatedly fall. We demonstrate that it is mostly their frailty and age that predicts mortality. A lower risk of mortality was seen in women. The median age in the cohort was 80 years, and frailty was clearly reflected in the high average Frailty Index of this cohort, with either moderate or severe frailty present in 441 patients (66.5%). A previous analysis of this cohort showed that the observed risk of major bleeding is similar to the large RCT’s [4]. In accordance with the recent meta-analyses, patients with atrial fibrillation in our cohort had higher frailty scores [15,18]. More than half of the atrial fibrillation patients were severely frail in this cohort, and as the authors of the meta-analyses state, perhaps atrial fibrillation should be seen as a cardiovascular marker of frailty [15]. In contrast to the recent meta-analyses, we studied a frail cohort of patients and not only patients with atrial fibrillation [15,18]. This allowed us to investigate the risk of using either antiplatelet agents or OAC on top of the high residual risk for adverse events that frail atrial fibrillation patients already possess. Our data are observational and should be interpreted with caution with regard to causality. However, they do not show an independent association with mortality and the use of OAC. This suggests that the caution and risk aversion that physicians may feel in (not) prescribing antithrombotic medication to frail older patients might be misplaced and bears the risk of undertreatment [1,2,8,15,25,26]. Higher chances that frail patients are not prescribed OAC as was seen in the European ESC-EHRA EORP-AF General Long-Term Registry could not be confirmed in our cohort, as we found a rate of OAC use for AF of 82% [13]. This is possibly explained by the well-organized Dutch Thrombosis Network. For as far as the cause of death was documented, most patients died as a consequence of infections and malignancies. There were only a few major or fatal bleedings. It is possible that especially within frail populations, the competing risks for mortality outweigh the risks of OAC and explain our observations within this Fall and Syncope Registry cohort. Arbel and colleagues performed a retrospective cohort study investigating the risk of mortality in moderate-to-high-risk AF patients [27]. Propensity matching of patients in whom a direct anticoagulant was initiated, compared to patients who remained without oral anticoagulation, showed a marked reduction in mortality for the DOAC-treated patients (HR 0.69, 95% CI 0.63–0.75) [26]. The START2-REGISTER study enrolled patients with AF of 85 years and older and showed that the all-cause mortality rate was higher in patients on vitamin K antagonists compared to those on DOAC, without a significant difference in major bleeding [28]. Similar to our findings, in the START2-REGISTER study, an association between frailty and mortality was found [28]. In this exploratory analysis of the association of antithrombotic medication and mortality within patients with atrial fibrillation only, our data suggest a survival advantage for those using OAC. This finding is in line with the recent meta-analyses and the retrospective study of Kim and colleagues [13,14,16,18]. The prescription of OAC or antiplatelet agents reflects daily practice, and treatment was not assigned at random. Patient-tailored decisions on whether to prescribe OAC or not could not be accounted for in our cohort. However, of the patients with atrial fibrillation in our cohort, only a minority of 11 patients were not treated with OAC, and 12 were using antiplatelet agents instead of OAC. It is very likely that specific patient factors lead to these decisions not to prescribe OAC, which could not be corrected for in this analysis. Indeed, the Frailty Index of these patients with atrial fibrillation on antiplatelet agents was on average 0.28 ± 0.08 higher than the mean Frailty Index of all atrial fibrillation patients, 0.26 ± 0.09, or the cohort overall mean, 0.23 ± 0.09. However, it would be of great value if more robust data from trials of prospective studies became available for frail patients with atrial fibrillation. The systematic review and meta-analysis of Chai-Adisaksopha and colleagues showed that the use of DOAC compared to warfarin was associated with significant reductions in fatal bleedings, cardiovascular mortality, and all-cause mortality [29]. Aforementioned studies involve anticoagulation naïve patients and do not address the question if patients who are on vitamin K antagonists will have the same benefits after switching to a DOAC. The FRAIL-AF study addressed this question specifically for frail older patients and found a higher incidence of clinically relevant non-major bleeding after switching from a well-adjusted VKA to a DOAC but no differences in major bleeding, fatal bleeding, or all-cause mortality within one year of follow-up [30]. Within the FRAIL-AF study, frailty was screened with the Groninger Frailty Indicator, which has an emphasis on daily functioning and independence [31]. Since atrial fibrillation is as well closely associated with other cardiovascular morbidities, obesity, and diabetes, an assessment of frailty with a CGA-based Frailty Index could describe the patients’ frailty in a more comprehensive manner.

Our study has its limitations and the results should be interpreted cautiously. It has an observational design with a continuous inclusion of patients and baseline data, but it is without systematic follow-up for other outcomes than mortality. Reliable information on initiation or discontinuation of antithrombotic treatment during follow-up was not available for this analysis, hence crossover may have occurred. Information on the time within therapeutic range in patients using vitamin K antagonists was not available. In the START2-REGISTER study, a lower percentage of time within therapeutic range (TTR) of vitamin K antagonists was observed in patients who died, and we cannot exclude that this is also a relevant factor within our cohort [28]. For patients with cognitive disorders or dementia, using a DOAC might overcome the difficulty of reaching an optimal TTR [32]. The majority of patients on oral anticoagulation used vitamin K antagonists in this cohort, and the results might be different for the DOACs. For this study, data on mortality were collected from the hospital records and potentially could be incomplete, especially on the cause of death. However, it is unlikely that death would have been overlooked, since the hospital periodically receives information on the dates of death from the National Personal Records Database. Most of the deaths occurred among the participants who visited the Fall and Syncope Clinic in the earlier years of the registry. The outcomes of the unadjusted risks should therefore be interpretated cautiously and mainly serve as an exploratory analysis prior to the cox regression survival analysis.

## 5. Conclusions

In this frail geriatric population, mortality was related to patients’ age, sex, and frailty. The use of either antiplatelet agents or oral anticoagulation was not independently associated with a higher risk of mortality. Therefore, a restriction toward prescribing antithrombotic medication to frail patients could be unjustified.

## Figures and Tables

**Table 1:** Baseline characteristics.

General Characteristics	Total n = 663	No Antithrombotic Medication, n = 298	Antiplatelet Agents, n = 221	Oral Anticoagulation, n = 144
Female, n (%)	438 (66.1)	213 (71.5)	142 (64.3)	83 (57.6)
Age in years, median (sd)	80 (6.5)	79 (6.7)	81 (6.0)	81 (6.5)
Number of drugs, median (sd)	7 (3.8)	5 (3.3)	8 (3.3)	8 (3.8)
Number of morbidities, median (sd)	10 (5.2)	8 (4.4)	10 (5.2)	12 (5.6)
Multiple falls per year, n (%)	584 (88.3)	263 (87.9)	196 (88.7)	125 (87.4)
Atrial fibrillation, n (%)	152 (22.9)	17 (5.7)	17 (7.7)	118 (81.9)
Hypertension, n (%)	432 (65.2)	163 (54.7)	164 (74.2)	105 (72.9)
Diabetes mellitus, n (%)	150 (22.6)	58 (19.5)	53 (24.0)	39 (27.1)
Creatinine clearance below 60 mL/min, n (%)	139 (21.0)	36 (12.1)	55 (24.9)	48 (33.3)
Heart failure, n (%)	64 (9.7)	7 (2.3)	20 (9.0)	37 (25.7)
Stroke in medical history, n (%)	149 (22.5)	13 (4.4)	95 (43.0)	41 (28.5)
Hypercholesterolemia, n (%)	172 (25.9)	49 (16.4)	75 (33.9)	48 (33.3)
Ischemic heart disease, n (%)	160 (24.1)	15 (5.0)	94 (42.5)	51 (35.4)
Geriatric features				
Frailty Index, mean (sd)	0.23 (0.09)	0.20 (0.08)	0.26 (0.08)	0.26 (0.09)
Moderately Frail, n (%)	182 (27.5)	83 (27.9)	66 (29.9)	33 (22.9)
Severe Frail, n (%)	259 (39.1)	73 (24.5)	107 (48.4)	79 (54.9)
Polypharmacy, n (%)	484 (73.0)	160 (53.7)	196 (88.7)	128 (88.9)
Orthostatic hypotension, n (%)	195 (29.4)	68 (22.8)	79 (35.7)	47 (32.6)
Post prandial hypotension, n (%)	208 (31.4)	91 (30.5)	67 (30.3)	50 (34.7)
Parkinsonism, n (%)	63 (9.5)	30 (10.1)	22 (10.0)	11 (7.6)
Gait disturbance, n (%)	345 (52.0)	153 (51.3)	118 (53.4)	74 (51.4)
ADL dependence, n (%)	158 (23.8)	59 (19.8)	57 (25.8)	42 (29.2)
iADL dependence, n (%)	262 (39.5)	103 (34.6)	94 (42.5)	65 (45.1)
Cognitive impairment				
MMSE < 26 points, n (%)	177 (26.7)	74 (24.8)	57 (25.8)	46 (31.9)
MoCA < 26 points, n (%)	380 (57.3)	172 (63.1)	125 (56.6)	83 (57.6)

**Table 2:** Causes of death.

	Total, n = 196 (29.6%)	No Antithrombotics, n = 71 (23.8%)	Antiplatelet Agents, n = 67 (30.3%)	Oral Anticoagulation, n = 58 (40.3%)
Infection, n (%)	36 (18.4)	8 (11.3)	17 (25.4)	11 (19.0)
Malignancy, n (%)	21 (10.7)	10 (14.1)	8 (11.9)	3 (5.2)
Cardiac, n (%)	14 (7.1)	3 (4.2)	6 (9.0)	5 (8.6)
Major bleeding, n (%)	12 (6.1)	8 (11.3)	2 (3.0)	2 (3.4)
Other, n (%)	28 (14.3)	8 (11.3)	8 (11.9)	12 (20.7)
Unknown, n (%)	85 (43.4)	34 (47.9)	26 (38.8)	25 (43.1)

**Table 3:** Unadjusted univariable analysis of mortality risks.

General Characteristics	Hazard Ratio	95% Confidence Interval	*p*
Female sex	0.44	0.31–0.62	<0.01
Age, risk per year	1.07	1.04–1.10	<0.01
Use of antithrombotic medication, as compared to no use			
Antiplatelet agents	1.55	1.04–2.31	0.03
Oral anticoagulation	2.14	1.38–3.32	<0.01
Number of prescription drugs	1.06	1.01–1.10	0.02
Number of morbidities	1.07	1.03–1.10	<0.01
Multiple falls per year	2.02	1.08–3.76	0.03
Atrial fibrillation	2.83	1.56–3.34	<0.01
Hypertension	1.19	0.83–1.70	0.20
Diabetes mellitus	1.06	0.71–1.59	0.43
Creatinine clearance 30–60 mL/min	1.68	1.16–2.43	<0.01
Creatinine clearance 15–30 mL/min	8.95	2.79–28.69	<0.01
Heart failure	3.40	2.01–5.73	<0.01
History of stroke	1.12	0.75–1.67	0.33
Hypercholesterolemia	1.18	0.81–1.73	0.23
Ischemic heart disease	1.98	1.36–2.88	<0.01
Geriatric features			
Moderate Frailty	2.47	1.50–4.05	<0.01
Severe Frailty	3.94	2.50–6.20	<0.01
Polypharmacy	1.15	0.78–1.70	0.27
Orthostatic hypotension	1.20	0.78–1.85	0.24
Post prandial hypotension	1.79	1.13–2.83	0.01
Parkinsonism	2.83	1.67–4.79	<0.01
Gait disturbance	1.54	1.09–2.18	0.01
ADL dependence	2.63	1.80–3.83	<0.01
iADL dependence	2.19	1.55–3.09	<0.01
Cognitive impairment			
MMSE < 26 points	1.66	1.14–2.40	0.01
MoCA < 26 points	1.41	0.94–2.12	0.06

**Table 4:** Adjusted cox regression survival analysis.

	Hazard Ratio	95% Confidence Interval	*p*
Use of antithrombotic medication, as compared to no use			
Antiplatelet agents	0.89	0.62–1.29	0.54
Oral anticoagulation	1.22	0.83–1.80	0.31
Female sex	0.46	0.34–0.62	<0.01
Age, risk per year	1.05	1.03–1.08	<0.01
Frailty Index	74.0	13.1–417.3	<0.01

## Data Availability

Data sharing was not part of the informed consent procedure for participants. Upon reasonable request, scientific cooperation and sharing a selection of data is possible.

## Supplementary Materials

The following supporting information can be downloaded at: https://www.mdpi.com/article/10.3390/jcm12237388/s1, Figure S1: Classification of Frailty in patients with Atrial Fibrillation; Table S1: Factors in the Frailty Index.
