# Supplementary material for: The Use of Oral Anticoagulation Is Not Independently Associated with Mortality in Frail Older Patients with Repeated Falls
Source: J Clin Med. 2023 Nov 29;12(23):7388. doi: 10.3390/jcm12237388 (PMC10706899; doi:10.3390/jcm12237388)
Supplement: Supplementary file 1 [file jcm-12-07388-s001.zip › jcm-2705328-supplementary.pdf]

Supplementary Table S1. Factors in the Frailty Index.

Mild Cognitive Impairment
Dementia
Syncope
Heart Failure
Ischemic Heart Disease
Peripheral arterial disease
Stroke
Multiple Stroke
Diabetes Mellitus
Liver cirrhosis
Hypertension
Anaemia
Renal Impairment, class 2 or up
Electrolyte abnormality
Hypothyroidism
Hyperthyroidism
Malignancy
Epilepsy
Schizophrenia
Morbus Parkinson or parkinsonism of other causes
Gait disorder
Visual Impairment
Auditory Impairment
Osteoporosis
Arthrosis
Chronic Obstructive Pulmonary Disease
Asthma
Obstructive Sleep Apnoea Syndrome
Mood disorder
Anxiety disorder
Falls
Obesitas
Underweight
Gout
Polyneuropathy
Polypharmacy
MMSE below 26 points
MoCA below 26 points
MNA lower than 12 points
ADL dependent
iADL dependent
Social status (living alone)

Explanatory note: All factors are assigned an equal weight if present in the patient. The index is calculated as the sum of factors, divided by 42. Polypharmacy is defined as using 6 or more different drugs.

Abbreviations. MMSE: Mini Mental State Examination, MoCA: Montreal Cognitive Assessment, MNA: Mini Nutritional Assessment, ADL: Activities of Daily Living, iADL: instrumental Activities of Daily Living.

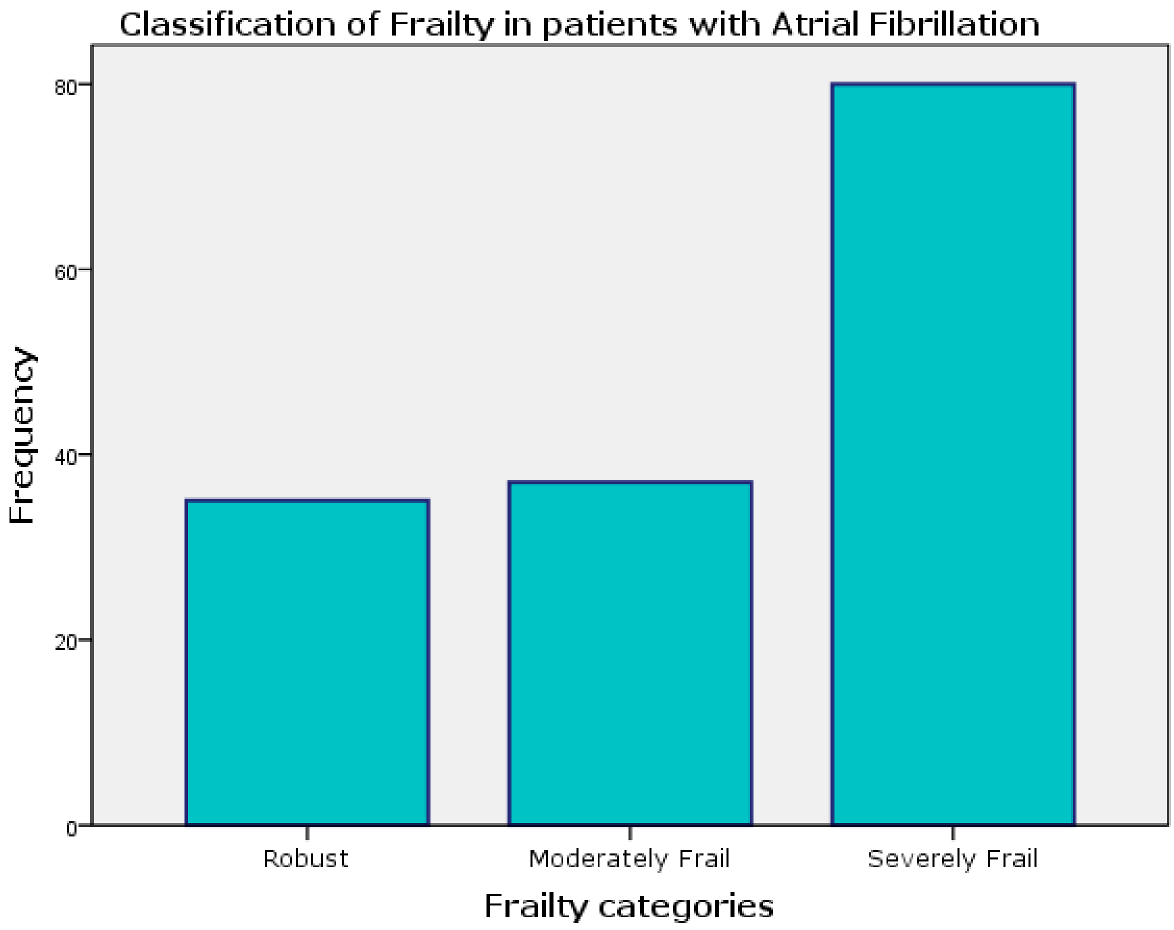

Supplementary Figure S1. Classification of Frailty in patients with Atrial Fibrillation.
